# NETWORK ANALYSIS OF INTEROCEPTIVE SENSIBILITY IN YOUNGER AND OLDER LIVER TRANSPLANT RECIPIENTS

**DOI:** 10.1093/geroni/igae098.3019

**Published:** 2024-12-31

**Authors:** JiYeon Choi, Seongmi Choi, Hanna Lee, Sang Hui Chu

**Affiliations:** Yonsei University College of Nursing, Mo-Im Kim Nursing Research Institute, Seoul, Republic of Korea; Yonsei University College of Nursing, Mo-Im Kim Nursing Research Institute, Seoul, Republic of Korea; Yonsei University College of Nursing, Mo-Im Kim Nursing Research Institute, Seoul, Republic of Korea; Yonsei University College of Nursing, Mo-Im Kim Nursing Research Institute, Seoul, Republic of Korea

## Abstract

Due to surgical advancements and post-transplant therapeutics, liver transplantation (LT) is increasingly viable for older adults with end stage liver conditions, such as hepatocellular carcinoma and non-alcoholic steatohepatitis. Effective post-LT symptom management is crucial for optimizing benefits of LT for older liver transplant recipients (LTRs). Interoceptive sensibility, which involves perceiving and being aware of internal body sensations, plays a vital role in symptom perception and response. Understanding interoceptive sensibility patterns across age groups may help tailor symptom management strategies for older LTRs. This study explored interoceptive sensibility in LTRs using network analysis on a sample of 200 Korean LTRs divided into two age groups: younger LTRs (< 60 years; n=122), and (2) older LTRs (≥ 60 years; n=78). Interoceptive sensibility was assessed using the Multidimensional Assessment of Interoceptive Awareness (version 2), comprising eight domains: noticing, not distracting, not worrying, attention regulation, emotional awareness, self-regulation, body listening and trusting. Network analysis examined centrality of nodes in the network for each age group. Bootstrapping (1,000 iterations) assessed network stability and accuracy. In younger LTRs, attention regulation reported the largest values for betweenness (rs=1.882), closeness (rs=1.585) and strength (rs=1.140). In older LTR, attention regulation domain reported the largest values for betweenness (re=1.666) and closeness (rs=1.907), while emotional awareness domain (rs=1.180) had the largest values for strength. Networks exhibited stability (correlation stability coefficient ≥ 0.5) and accuracy (small confidence interval). Addressing central interoceptive sensibility domains, particularly emotional awareness, may improve the tailored symptom management strategies for older LTRs, potentially enhancing post-LT outcomes.

